# The impact of COVID-19 on obstetrics and gynaecology trainees; how do we move on?

**DOI:** 10.52054/FVVO.13.1.004

**Published:** 2021-03-31

**Authors:** R Mallick, F Odejinmi, M Sideris, E Egbase, M Kaler

**Affiliations:** Princess Royal Hospital, Brighton and Sussex University Hospitals NHS Trust, Lewes Road, Haywards Heath, RH16 4EX, UK; Whipps Cross Hospital, Barts Health NHS Trust, Whipps Cross Road, Leytonstone, London, E11 1NR, UK

**Keywords:** COVID-19, coronavirus, training

## Abstract

**Background:**

Obstetrics and Gynaecology (O&G) is an evolving specialty that encompasses women’s health at its core. The COVID-19 pandemic has caused significant patient care challenges, however simultaneously it has resulted in the interruption of clinical training and cessation of all elective work. Our primary aim was to assess the impact of the pandemic on the experiences of O&G trainees.

**Methods:**

An email invite was sent to all 127 O&G trainees in Kent, Surrey and Sussex (KSS), inviting them to participate in an anonymous 33-question survey. The survey data was collected and analysed over a 4-week period.

**Results:**

Of the 127 trainees sent the survey, 87 responded (69%). 39% and 75% of trainees agreed that the pandemic had a negative impact on their overall physical and mental wellbeing respectively. 43% agreed that the COVID-19 pandemic had adversely affected their obstetric training experience whilst almost all trainees stated a significant negative impact on benign gynaecology surgical training. Reassuringly, over 80% were positive they would recover from the negative impacts of COVID-19.

**Conclusions:**

It is evident that COVID-19 has impacted O&G trainees in several ways. Whilst we face uncertain times, we must firstly ensure the physical and mental well-being of all trainees. It is encouraging that non-emergency consultations and benign surgery are being restarted nationwide and whilst this will inevitably help with re-booting surgical training, we must also think “outside” the box and utilise other modes of teaching and training to safeguard learning whilst mitigating against the negative impacts of subsequent waves.

## Background

The COVID-19 pandemic has overwhelmed healthcare services worldwide and its impact continues to be felt particularly within benign gynaecology (Mallick et al., 2020). The clinical challenges and potential long-term consequences with regards to patient care, healthcare processes and pathways have been detailed in the wider literature and guidance is in place to aid the “recovery process” (Odejinmi et al., 2020; NICE, 2020). An area, somewhat overlooked, that does necessitate in-depth exploration are the long-lasting effects that this worldwide pandemic is having and will continue to have on the training of the future medical workforce. Training has been significantly disrupted with many junior trainees deployed to support front line services. Furthermore, training opportunities available in the outpatient and surgical setting as well as simulation and skills and drills have diminished with the cessation of face to face teaching, clinical consultations, non-emergency surgery and outsourcing of elective surgeries to the independent sector. For many the priority has shifted from training to much needed service provision. This had undoubtedly taken a large toll on the physical and mental wellbeing of our trainees and the consequences of this are much more difficult to assess and mitigate against.

The aim of this survey was to assess the wider impact of the COVID-19 pandemic on the experiences of obstetrics and gynaecology (O&G) trainees. The experiences explored within this survey include training opportunities, surgical practices and the effects of these unprecedented times on the mental and physical wellbeing of the trainees as well as thoughts from trainees on how best to move forward out of the pandemic and optimise training opportunities.

## Methods

All O&G trainees within the Kent, Surrey and Sussex (KSS) region were sent an email invite to undertake an anonymous 33-question survey. The response range included strongly disagree, disagree, neither agree nor disagree, agree, strongly agree. Informed consent was gained via the initial email invite and the data was collected over a 4-week period. Data was analysed using SPSS version 27. Thematic analysis of the data including trainee comments was also undertaken.

## Results

### 

**Figure 1:**
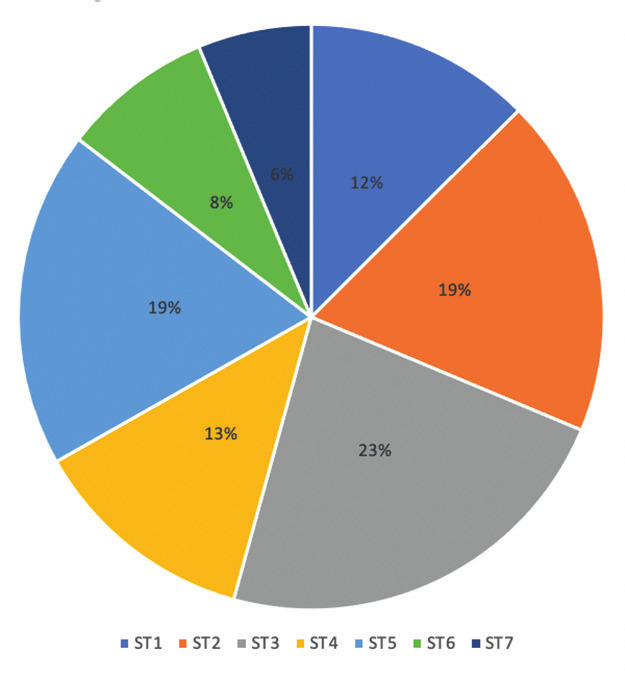
— Training level.

Of the 127 O&G trainees currently in programme within the KSS region, 87 responded giving a 69% response rate. The training level of the respondents is summarised in Figure 1.

### Health and wellbeing

39% (34/87) and 77% (67/87) of trainees agreed or strongly agreed that the COVID-19 pandemic had a negative impact on their physical and mental wellbeing respectively. 60% (52/87) had adequate access to COVID-19 polymerase chain reaction (PCR) antigen testing when required. Over 90% (79/87) felt they had adequate access to COVID-19 antibody testing. 75% (66/87) felt they had adequate access to personal protective equipment (PPE) when covering obstetrics compared to 83% (72/87) when covering gynaecology, however only 56% (49/87) of all trainees felt they had adequate training to use the PPE. 50% (43/87) of those questioned had taken time off work due to a COVID-19 related issue. The average time taken off work was 9 days (range 1-90). Of the 36 trainees who had a COVID-19 PCR antigen test, 14% (5/36) tested positive. Of 55 trainees to have undertaken a COVID-19 antibody test, 7% (4/55) tested positive.

### Work and training

Over 80% (72/87) of trainees felt their overall working pattern had altered due to COVID-19 and 11/87 (13%) were deployed outwith O&G to aid the COVID response. The majority were deployed to intensive care and of those deployed, 73% (8/11) did not feel clinically competent to do so.

43% (37/87) agreed or strongly agreed that the COVID-19 pandemic had negatively affected their obstetric training experience compared to almost 99% (86/87) who felt their benign gynaecology surgical training experience had been negatively affected. 46% (40/87) and 93% (81/87) of trainees agreed or strongly agreed that their antenatal clinic and gynaecology clinic experiences respectively, had been negatively affected. 47% (41/87) of trainees felt virtual obstetric and gynaecology clinics did not adequately compensate for the training experiences normally gained from face to face clinics. 93% (81/87) of O&G KSS trainees felt their educational activities had been negatively affected due to COVID-19.

79% (69/87) felt concerned on the overall impact of COVID-19 on their training, with 56% (49/87) stating that they felt their Annual Review of Competence Progression (ARCP) outcome and/ or training progression may be adversely affected. 40% (35/87) felt training time should be extended for all to compensate for COVID-19. 84% (73/87) were positive they would recover from the negative impacts of COVID-19. Table I summarises the trainee responses to the wellbeing and training questions.

**Table I:** Trainee responses regarding wellbeing and training.

	Strongly agree/agree (%)	Neither agree nor disagree (%)	Strongly disagree/disagree (%)
Wellbeing			
The COVID-19 pandemic has affected your physical wellbeing	39	20	41
The COVID-19 pandemic has affected your mental wellbeing	77	5	18
Training			
COVID-19 has negatively affected your obstetric clinical training experience	43	16	41
COVID-19 has negatively affected your obstetric antenatal training experience	46	15	39
COVID-19 has negatively affected your obstetric ultrasound experience	61	26	13
COVID-19 has negatively affected your gynaecology outpatient training experience	93	3	4
COVID-19 has negatively affected your emergency gynaecology surgical training experience	84	9	7
COVID-19 has negatively affected your benign gynaecology surgical training experience	99	1	0
My educational opportunities have been negatively affected by COVID-19	93	5	2
I am concerned about the impact of COVID-19 on my overall training	79	15	6
COVID-19 will likely have a negative impact on my training progression/anticipated ARCP outcome	56	29	15

**Figure 2:**
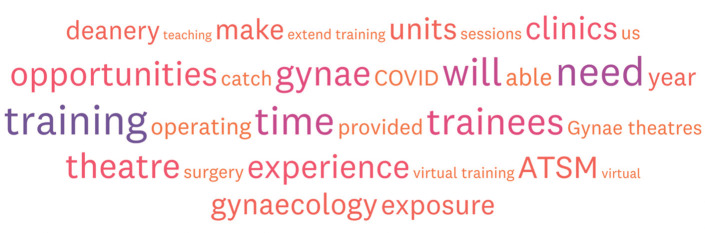
— How trainees can be supported moving forward.

**Figure 3:**
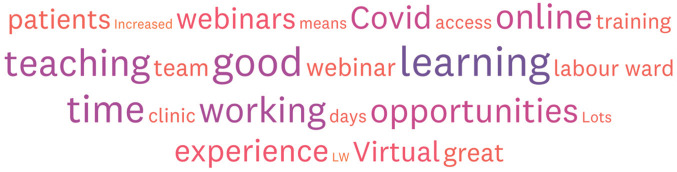
— The positive outcomes.

Figure 2 summarises the main themes when trainees were asked to comment on how they could best be supported to minimise the impact of the pandemic moving forward. Figure 3 summarises the positive opportunities that trainees feel have arisen from this pandemic.

## Discussion

The results of this survey confirm our initial assumptions on the negative effects of the COVID- 19 pandemic on O&G trainees (Warhadpande et al., 2020; He et al., 2020). We acknowledge this is a small survey sample undertaken in the south of England and a nationwide survey would glean more information, however the pandemic appears to be affecting the whole of UK in a very similar fashion across all medical specialities. We also acknowledge the potential response bias in this small sample as those most negatively impacted are more likely to respond. Our 69% response rate is reassuring; however, a larger national study would be greatly beneficial to fully assess the different challenges nationwide. The challenges highlighted in this survey and the strategies suggested to safeguard trainees are certainly generalisable to the whole of the UK and Europe. Similar surveys from other specialities in the UK and beyond highlight much the same concerns (Caruana et al., 2020; IIonzo et al., 2021; Sneyd et al.,2020).

Given these significant findings and how protracted the “first-wave” of the pandemic was and with the “second wave” well underway, one can assume these effects will be long-lasting and we must consider how best to protect and safeguard training in these subsequent waves, which may be more protracted and challenging than the first.

The redeployment of trainees to areas of clinical need, such as intensive care, was unavoidable in the first wave. However, the Royal College of Obstetricians and Gynaecologists (RCOG) is clear in their views that the maternity workforce should not be redeployed unless there is no other viable option (RCOG, 2020). Our results show a redeployment rate of 13% with 73% (8/11) of respondents stating they did not feel clinically competent to work in the areas they were redeployed. This is a challenging issue and questions the clinical competency of junior doctors deployed to such areas and the knock-on negative effects this could have on patient safety as well as staff wellbeing.

In the event of further redeployment, clinical education is essential to tackle this issue with current literature demonstrating that a collaborative approach particularly with the multidisciplinary team is necessary. Within the medical profession, doctors have many transferable skills relevant to clinical care which should be harnessed (Coughlan et al., 2020). The Faculty of Intensive Care Medicine (FICM) produced a document to support local providers in identifying training needs, resources and support for deployed staff (FICM, 2020). The document, which has been endorsed by the Royal College of Surgeons (RCS) highlights both the clinical and non-clinical skills needed to work safely in the critical care setting during the pandemic. This forms a framework upon which an education programme for deployed staff can be based on. In addition, this document signposts users to further training resources. Establishing training sessions for specific clinical skills needed to safely work in the areas of redeployment can involve peer to peer learning, webinars and face to face teaching of clinical skills. Collins and colleagues describe a successful trainee led multimodal education programme established in response to the pandemic (Collins et al., 2020).

It is also key that we consider the overall psychological and physical burden that this pandemic had had on trainees particularly with regards to stress, burnout and mental wellbeing. Our survey highlighted that over one third of trainees felt the COVID-19 pandemic had negatively affected their physical wellbeing and over two thirds of those surveyed felt a negative impact on their mental wellbeing. This has been highlighted in other studies which document the negative psychological impacts of the pandemic on patients and healthcare professions (Wang et al., 2020; Tan et al., 2020). It is also prudent to acknowledge the impact of poor safety provision and the lack of training in PPE, especially at the beginning of the pandemic, has had on the wellbeing of medical staff with only 50% of the participants in this survey believing they had adequate training in PPE. It is imperative therefore, that PPE training is added to mandatory hospital training as part of a safe induction programme with ongoing yearly updates. A recent invaluable article in the BMJ entitled “Doctors’ wellbeing: self-care during the COVID-19 pandemic” also highlighted the importance of NHS staff wellbeing during the pandemic (Unadkat and Farquhar, 2020). They emphasised the importance of regular rest, breaks and sleep as well as the need for hospital trusts to mobilise their psychological services to provide direct support to trainees where needed, thus providing practical tools to improve wellbeing. This is fundamental to safeguard the wellbeing of trainees in general but is particularly essential during these unprecedented times.

As expected and highlighted in our survey both obstetrics and gynaecology training have been negatively impacted. Predictably the biggest concerns from trainees are around gynaecological surgical training with over 99% agreeing this had been negatively impacted. The decision to postpone all non-urgent elective operations resulted in an estimated 516,000 operations being cancelled (COVIDSurg Collaborative, 2020). The triaging of operations based on patient safety and clinical need has manifested in a slow re-introduction of benign gynaecology services, less operating time and capacity issues which have resulted in significantly less training opportunities. This has particularly impacted senior trainees completing surgical Advanced Training Skills Modules (ATSMs). As a result, we must prioritise training opportunities for those who need it most and this should ideally be done at a deanery level following ARCPs. Such trainees should be strategically placed in hospital trusts less impacted with focused surgical training targets reviewed on a regular basis.

Furthermore, there is also a need to now think outside the box and explore other avenues of surgical training such as simulation and virtual training and begin to embed these into the O&G curriculum moving forward. This is the only way to truly safeguard surgical training in the event of further pandemic waves and subsequent cessation again of benign services.

All trainees should have an individualised trainee focused plan in the months and years following COVID-19 to highlight areas which have been negatively impacted by this pandemic and training plans put in place to support gaining missing competencies. This was a common theme in the feedback from trainees undertaking this survey. The option to extending training due to COVID-19 without negative consequences should be available to every trainee if they feel this may be required following detailed discussion with their educational supervisor and training programme directors (TPDs). 40% of trainees in this survey felt training time should be extended for all to compensate for the lost training as a consequence of COVID-19.

The overall paradigm shift in clinical working from face to face to digital via virtual clinics, Zoom meetings and Team presentations must be accompanied with a shift in the approach to training by trainers and the expectations of trainees. Using digital platforms can improve access, flatten hierarchies within the teaching environment and has the benefit of allowing people to catch up at a suitable pace. Our data shows a significant number of trainees have spent time self-isolating or quarantining and digital learning enables such trainees to feel part of a community of practice, something which has be extremely beneficial and will continue to be beneficial in the event of subsequent waves and particularly to those trainees who are shielding.

Virtual learning has undoubtedly been the single most positive consequence of this pandemic and this is clearly highlighted in our survey especially in the trainee comments. Trainees value the flexibility it offers and strongly feel that these new ways of learning should be continued post pandemic and are an exciting platform to develop from. Whilst definitely a positive, these sessions are increasingly being offered out of standard work hours and caution is therefore essential to ensure this does not negatively impact trainee wellbeing, work life balance and worsen burnout.

## Conclusion

Our survey highlights the negative impact of the COVID-19 pandemic on O&G trainees. There is undoubtedly paramount concern with the possibility of further re-deployment with the ongoing second wave and potential futures waves. As a specialty we must consider and address these concerns with trainers and trainees. It is clear that the physical and mental wellbeing of trainees has been adversely affected during the pandemic. It is our responsibility to ensure support is available and easily accessible. Sleep and adequate rest are essential and exercise if possible, should be encouraged.

By far, cessation of elective gynaecological surgery has had the most negative impact on surgical training. It is vital that training needs are once again recognised and addressed in light of the impact of COVID-19. All trainees should meet with education supervisors and/or TPDs and create an individualised training plan for the next training year post COVID-19 to aid recovery and highlight the key training needs. A potential need for further training should be highlighted in this plan. Despite the negativity surrounding the impact of COVID-19 on training, we are faced with an exciting opportunity to develop how we deliver training long term and utilise alternative training methods in particular virtual and simulated training. Hands-on surgical training cannot be replaced, however whilst non- emergency services are slowly re-introduced, we must be innovative to ensure teaching and training continues in O&G and safeguard for the future.
